# Incidence rate trends of histological subtypes of nasopharyngeal carcinoma in Hong Kong

**DOI:** 10.1038/sj.bjc.6603413

**Published:** 2006-10-10

**Authors:** L A Tse, IT-S Yu, O W-K Mang, S-L Wong

**Affiliations:** 1 Department of Community and Family Medicine, The Chinese University of Hong Kong, Hong Kong, China; 2 Hong Kong Cancer Registry, Hospital Authority, Hong Kong, China

**Keywords:** nasopharyngeal neoplasm, incidence, trend, age–period–cohort models, aetiology

## Abstract

The overall decline in incidence rate of nasopharyngeal carcinoma in Hong Kong during 1988–2002 was limited primarily to a decrease in keratinising carcinoma, which could be explained by the decline in cigarette smoking. Genetic and Epstein–Barr virus interactions may explain the relatively stable incidence rate of non-keratinising carcinoma.

Nasopharyngeal carcinoma (NPC), although rare in most parts of the world, shows high rates among Cantonese in the central region of Guangdong Province in Southern China and Hong Kong, and among native people of Sarawak, Borneo (Yu and Yuan, 2002; Devi *et al*, 2004). Nasopharyngeal carcinoma incidence among Hong Kong Chinese are still the highest reported in the world, despite an appreciable decrease over the years (Lee *et al*, 2003). An overall decline in the NPC incidence among Chinese Americans in Los Angeles County and the San Francisco Metropolitan area was observed during the period 1992–2002 (Sun *et al*, 2005). The decline was primarily limited to tumours of the World Health Organization (WHO) type I (keratinising squamous cell carcinoma), whereas a relatively stable incidence rate of WHO type III tumours (undifferentiated carcinoma) was observed throughout that period. We have examined the time trends of NPC histological subtypes in Hong Kong, as these have not previously been reported, to see if trends similar to those among Chinese Americans could be observed.

## MATERIALS AND METHODS

Newly diagnosed NPC cases (excluding sarcoma, melanoma and carcinosarcoma) during the period 1988–2002 were identified from the Hong Kong Cancer Registry. For the present purposes, all NPC cases were regrouped into two histological types according to the WHO 1991 classification (Shanmugaratnam and Sobin, 1991), namely keratinising squamous cell carcinoma (equivalent to WHO Type I) and non-keratinising carcinoma (pooling together WHO Type II non-keratinising and Type III undifferentiated carcinoma in the old terminology) (Shanmugaratnam and Sobin, 1978, 1991). Those without pathological verification were regarded as of unknown tumour type. Misclassification owing to regrouping should be minimal as the basis of histology and histological subtype of each NPC case was recorded routinely by the Hong Kong Cancer Registry.

Population data during the corresponding period were obtained from the Hong Kong Census and Statistics Department. Mid-year population data were employed in the calculation of the incidence rates. We calculated the age-standardised annual incidence rate for each NPC subtype by the direct method using the WHO standard population (1980) as reference. Annual percentage change (APC) in incidence rate was calculated by using nonlinear regression under the assumption of same rate of change throughout the study period. Multiple Poisson regressions were fitted for each gender to examine the period and birth cohort effects on histology-specific NPC incidence rates using SAS procedure GENMOD after adjusting for age (SAS Institute, 1993). Three models – age alone, age–period and age–cohort were generated. A model including all three time variables was not computed because the use of a full age–period–cohort model may involve serious methodological difficulties (Clayton and Schifflers, 1987a, 1987b). Goodness of fit was assessed by the deviance; the closer the deviance to the degrees of freedom, the better the model fit (Clayton and Schifflers, 1987a, 1987b).

## RESULTS

**Figure 1:**
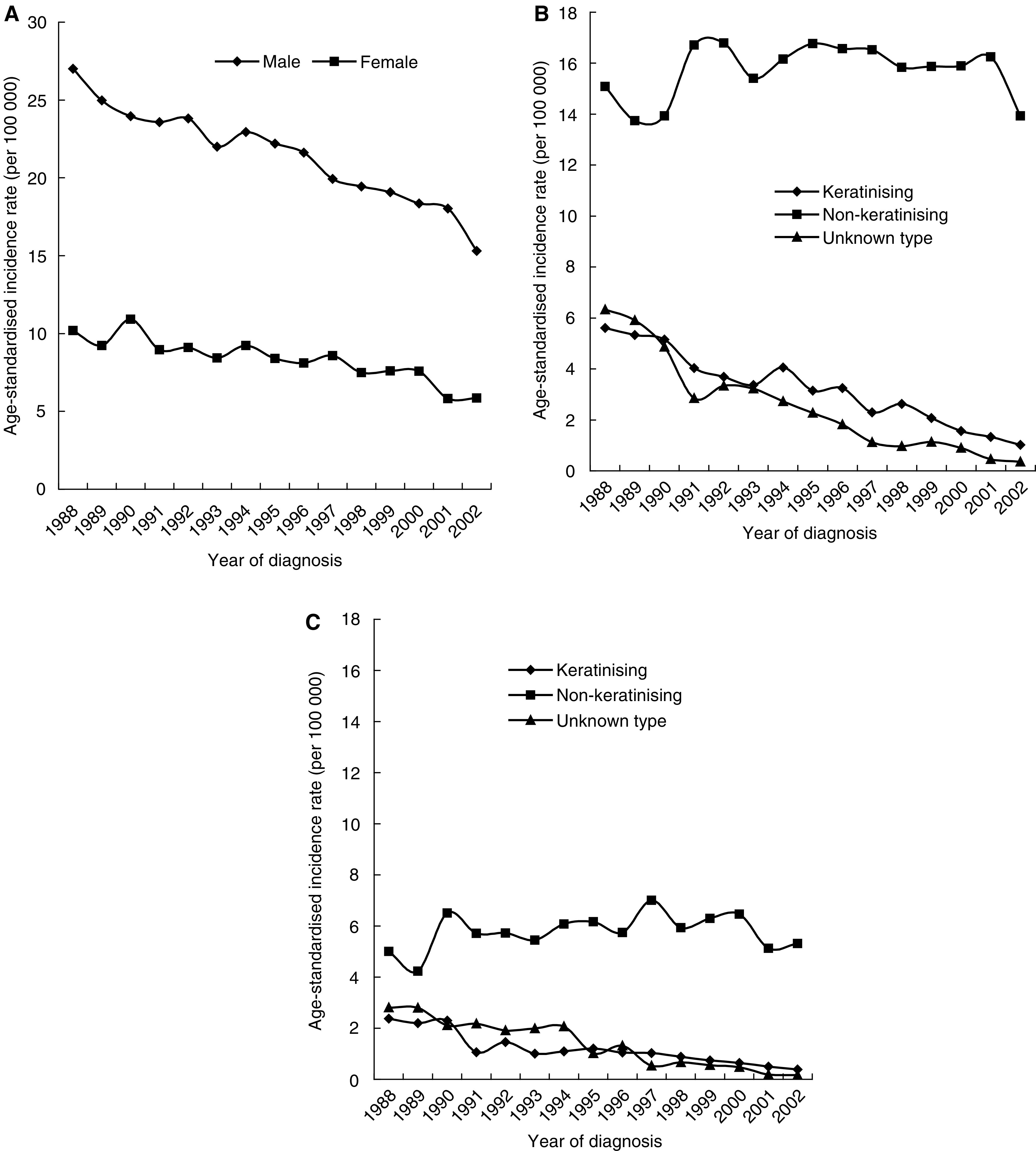
Age-standardised incidence rates of overall NPC among Hong Kong males and females (**A**) and by histological type among males (**B**) and females (**C**) during 1988–2002, using WHO 1980 standard population as reference.

During the period 1988–2002, a total of 16 333 new cases of NPC were diagnosed (11 838 males and 4495 females). Nine cases with unknown age were excluded from further analysis. The age-standardised incidence rate decreased for both males (APC=−3.1%, 95% confidence interval (CI): −2.6, −3.6) and females (APC=−3.3%, 95% CI: −2.2, −4.3), with the male to female ratio ranging between 2.2 and 3.1 (Figure 1A).

The proportion of NPC cases verified by histology improved progressively from 76% in 1988 to 97.3% in 2002, being 84% in 1992. The proportion of non-keratinising carcinoma in 2002 (90.4%) was substantially higher than that in 1988 (54.3%), whereas a lower proportion of keratinising carcinoma was diagnosed in the more recent years (6.9% in 2002 *vs* 21.5% in 1988). More than 99% of non-keratinising carcinomas were classified as undifferentiated carcinoma throughout the study period.

As shown in Figure 1B and C, the overall decline in NPC incidence rate was mainly due to the decline in the rates of keratinising and unknown type NPC. The trend for non-keratinising tumours was fairly horizontal in both genders, except for an apparent slight decrease in 2002 in males.

Deviance statistics from Table 1 suggested that the age–cohort model fitted better for keratinising tumours, whereas the age–period model fitted best for the unknown type tumours, but the differences were small. For non-keratinising carcinoma, only the age–period model was statistically significant.

**Figure 2:**
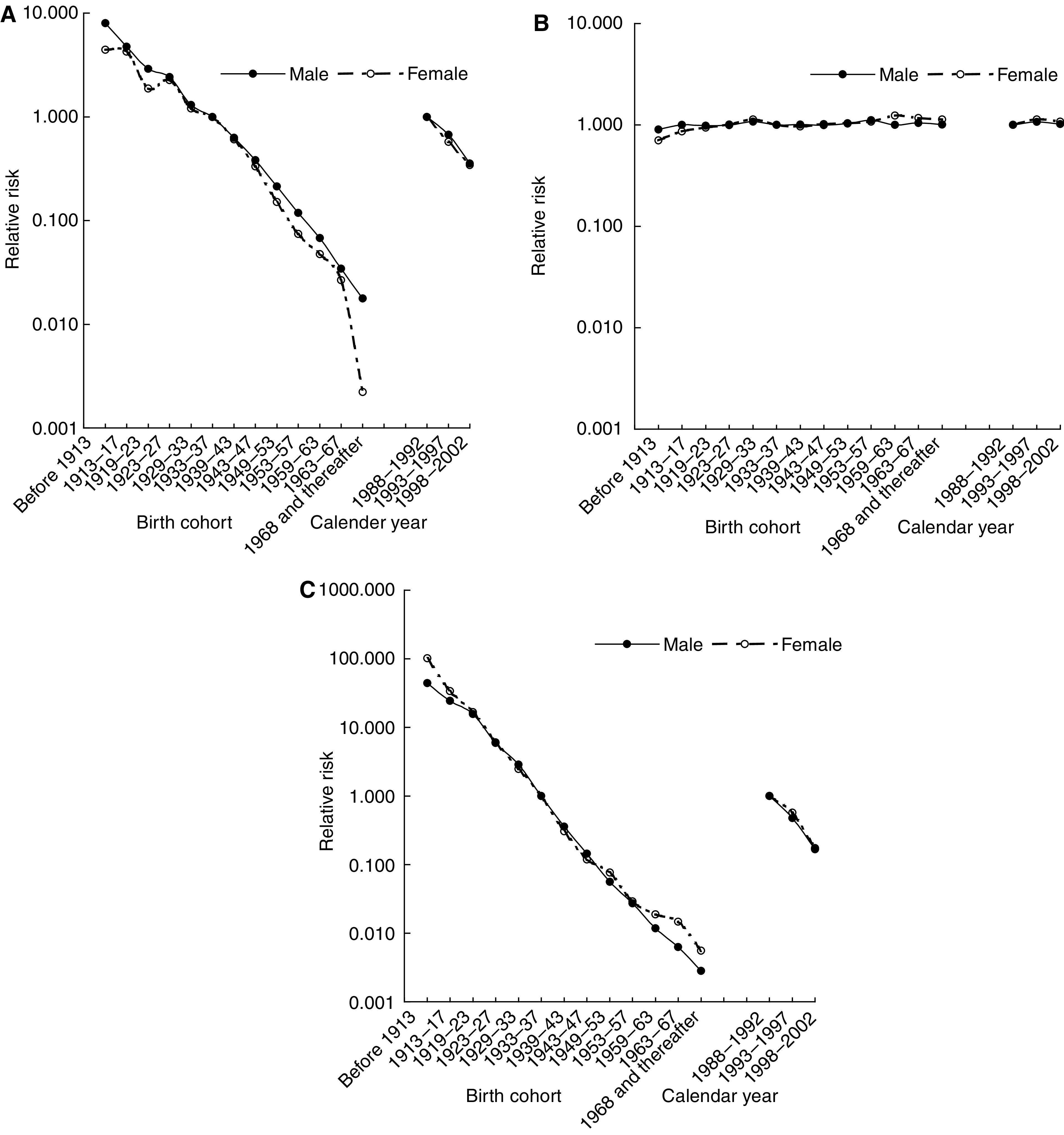
Cohort and period effects obtained from age–period and age–cohort analyses for keratinising carcinoma (**A**), non-keratinising carcinoma (**B**) and unknown type NPC (**C**) during the period 1988–2002 (in log scale).

The relative risks (RRs) in different calendar periods and birth cohorts of each subtype are shown in Figure 2. After the adjustment for age, the RR of keratinising carcinoma dropped dramatically from the period 1988–1992 to 1998–2002 by 60%. The decrease was more marked for unknown type tumours (80% drop). Very small increased period effects for non-keratinising tumours were observed in the last two periods during 1993–1997 (RR=1.07 for males and 1.13 for females) and 1998–2002 (RR=1.02 for males and 1.08 for females). Relative risks of consecutive birth cohorts for keratinising and unknown type tumours declined appreciably, with more rapid declines in the earlier birth cohorts in both genders. No clear birth cohort effects were observed for non-keratinising tumours.

## DISCUSSION

The decline in overall NPC incidence in Hong Kong during the period 1988–2002 was limited primarily to the decrease in keratinising and unknown type tumours. As shown in Table 2, compared with Chinese Americans (1992–2002 with a total of 359 male cases) and using the US 2000 standard population as reference, age-standardised rates were much higher among Hong Kong males for non-keratinising carcinomas, but there were no obvious differences in keratinising carcinoma (Sun *et al*, 2005). The pattern of time trends of the corresponding subtypes of NPC was quite similar between Chinese American and Hong Kong Chinese males.

The similar time trends observed among Hong Kong Chinese and Chinese Americans for different histological NPC types, the strong birth cohort effects observed in keratinising tumours and the lack of birth cohort effects and minimum period effects in non-keratinising tumours are relevant to certain aetiological hypotheses.

The age–cohort model fitted well for keratinising tumours in both genders, suggesting that changing exposures to environmental risk factors in early life operated in successive cohorts. The decreasing risk in recent periods, although statistically significant, was probably a reflection of birth cohort rather than a genuine period effect. Any possible mis-/re-classification would have resulted in significant increasing trend and period effects in non-keratinising tumours, which was not observed in this study. The progressive declines in more recent generations could be explained by the gradual reduction of population exposures to environmental risk factors. The association of keratinising tumours with Epstein–Barr virus (EBV) has long been controversial (Spano *et al*, 2003), but a relationship with tobacco smoking has been found in several studies (Vaughan *et al*, 1996; Spano *et al*, 2003; Sun *et al*, 2005). The overall smoking prevalence which has steadily decreased in Hong Kong since the mid-1970s for both genders among Hong Kong Chinese (Tobacco Control Office, 2006) could well explain the declining trend of keratinising tumours, allowing for a 10-year lag after exposure to cigarette smoking. Findings in Chinese Americans and our study support that changes in smoking patterns among males may, to some extent, account for the trends of keratinising tumours, although the relationship among females was not as strong as that among males. Consumption of Cantonese-styled salted fish containing lots of nitrosamines during weaning and childhood was believed to be the most important non-viral environmental risk factor for NPC (Yu, 1991; IARC, 1997), whereas no appreciable association with either keratinising or non-keratinising tumours was found.

Sporadic NPC is usually of the well-differentiated type whereas familial disease is poorly differentiated (McDermott *et al*, 2001). The lack of birth cohort effects for non-keratinising tumours in Hong Kong Chinese suggests that it is not related to environmental exposures or that environmental exposures associated with it have remained stable through generations. Genetic factors are likely to play a major role here, as implied by the marked differences in the incidence rates between Hong Kong Chinese and Chinese Americans, the former being mainly Cantonese originating from Guangdong Province, whereas the latter have more diverse origins.

To date, EBV seems to be exclusively associated with undifferentiated carcinoma in the nasopharynx (McDermott *et al*, 2001). However, this virus is ubiquitous and exposure to it has probably remained stable through recent generations. Gene and EBV interactions would offer the most likely aetiological agents.

In conclusion, the overall decline in NPC incidence rate was limited primarily to keratinising tumours, consistent with the decline in smoking. The relatively stable incidence of non-keratinising carcinoma and the lack of birth cohort effects are consistent with the role of gene and EBV interactions.

## Figures and Tables

**Table 1:** Results of poisson regression models for nasopharyngeal carcinoma incidence by histological type in Hong Kong, 1988–2002

			**Keratinising tumour^a^**		**Non-keratinising tumour^a^**		**Unknown type**	
**Variables in the model**	**DF**	**Gender**	**Deviance**	**Deviance/DF**	**Deviance**	**Deviance/DF**	**Deviance**	**Deviance/DF**
Intercept	32	Male	1981.8	61.9	8645.6	270.1765	1879.6	58.7
		Female	617.8	19.3	2626.6	82.0808	850.4	26.6
Age	22	Male	297.7	13.5	24.5	1.1143	587.6	26.7
		Female	145.4	6.6	31.9	1.4518	309.6	14.1
Age+period	20	Male	14.5 (*P*<0.001)	0.7	18.3 (*P*=0.045)	0.9168	22.0 (*P*<0.001)	1.1
		Female	32.8 (*P*<0.001)	1.6	25.0 (*P*=0.0318)	1.2515	32.2 (*P*<0.001)	1.6
Age+cohort	10	Male	7.5 (*P*<0.001)	0.8	13.7 (*P*=0.55)	1.3721	14.0 (*P*<0.001)	1.4
		Female	9.9 (*P*<0.001)	1.0	21.3 (*P*=0.56)	2.133	24.1 (*P*<0.001)	2.4

DF=degree of freedom.

*P*-values in parenthesis are for model improvements with the addition of the parameters compared with the age alone models.

a WHO 1991 classification.

**Table 2:** Comparisons of incidence rates of histological subtypes of nasopharyngeal carcinoma among males between Hong Kong Chinese (11 838 cases) and Chinese Americans (359 cases) based on WHO 1991 classification (using US 2000 standard population as reference) (Sun *et al*, 2005)

	**Keratinising**	**Non-keratinising**	**Unknown type^a^**	**All types**
1992	4.75 (3.29)	20.82 (4.85)	4.15 (6.44)	29.72 (14.58)
1993	4.17 (6.13)	19.23 (3.93)	3.88 (5.08)	27.28 (15.14)
1994	5.20 (3.92)	20.07 (2.39)	3.32 (2.94)	28.59 (9.25)
1995	4.09 (2.82)	21.06 (3.60)	2.85 (3.64)	28.00 (10.06)
1996	4.15 (5.15)	21.02 (3.58)	2.38 (6.21)	27.55 (14.94)
1997	2.92 (3.63)	20.83 (4.28)	1.38 (4.85)	25.14 (12.76)
1998	3.36 (3.04)	19.78 (6.03)	1.26 (5.34)	24.39 (14.41)
1999	2.56 (1.95)	20.03 (6.49)	1.43 (1.94)	24.02 (10.38)
2000	1.94 (1.25)	20.23 (3.75)	1.16 (4.06)	23.33 (9.06)
2001	1.71 (2.11)	20.36 (5.12)	0.65 (2.12)	22.72 (9.35)
2002	1.38 (1.14)	17.46 (4.05)	0.53 (3.20)	19.37 (8.39)

In parenthesis ( ) are the incidence rates of Chinese American.

a All cases in the American series were microscopically confirmed and the ‘unknown type’ among Chinese American meant the specified type unknown.
